# A Rare Case of Cerebral Venous Thrombosis and Disseminated Intravascular Coagulation Temporally Associated to the COVID-19 Vaccine Administration

**DOI:** 10.3390/jpm11040285

**Published:** 2021-04-08

**Authors:** Vincenzo D’Agostino, Ferdinando Caranci, Alberto Negro, Valeria Piscitelli, Bernardino Tuccillo, Fabrizio Fasano, Giovanni Sirabella, Ines Marano, Vincenza Granata, Roberta Grassi, Davide Pupo, Roberto Grassi

**Affiliations:** 1 Neuroradiology Unit, PO Ospedale del Mare, ASL NA1, via Enrico Russo, 80147 Naples, Italy; vincenzo-dagostino@libero.it (V.D.); alberto.negro@hotmail.it (A.N.); valeria.piscitelli@libero.it (V.P.); fabriziodoc@gmail.com (F.F.); gianni.sirabella6@gmail.com (G.S.); 2 Department of Medicine of Precision, School of Medicine, “Luigi Vanvitelli” University of Campania, 80147 Naples, Italy; roberta.grassi89@gmail.com (R.G.); dave.dp93@gmail.com (D.P.); roberto.grassi@unicampania.it (R.G.); 3 Cardiology Unit, PO Ospedale del Mare, ASL NA1, via Enrico Russo, 80147 Naples, Italy; bernardino.tuccillo@aslnapoli1centro.it; 4 Radiology Unit, PO Ospedale del Mare, ASL NA1, via Enrico Russo, 80147 Naples, Italy; ines.marano@tiscali.it; 5 Division of Radiology, “Istituto Nazionale Tumori IRCCS Fondazione Pascale—IRCCS di Napoli”, I-80131 Naples, Italy; v.granata@istitutotumori.na.it; 6 Italian Society of Medical and Interventional Radiology (SIRM), SIRM Foundation, 20122 Milan, Italy

**Keywords:** COVID-19, cerebral venous thrombosis, intravascular coagulation, vaccine

## Abstract

Globally, at the time of writing (20 March 2021), 121.759.109 confirmed COVID-19 cases have been reported to the WHO, including 2.690.731 deaths. Globally, on 18 March 2021, a total of 364.184.603 vaccine doses have been administered. In Italy, 3.306.711 confirmed COVID-19 cases with 103.855 deaths have been reported to WHO. In Italy, on 9 March 2021, a total of 6.634.450 vaccine doses have been administered. On 15 March 2021, Italian Medicines Agency (AIFA) decided to temporarily suspend the use of the AstraZeneca COVID-19 vaccine throughout the country as a precaution, pending the rulings of the European Medicines Agency (EMA). This decision was taken in line with similar measures adopted by other European countries due to the death of vaccinated people. On 18 March 2021, EMA’s safety committee concluded its preliminary review about thromboembolic events in people vaccinated with COVID-19 Vaccine AstraZeneca at its extraordinary meeting, confirming the benefits of the vaccine continue to outweigh the risk of side effects, however, the vaccine may be associated with very rare cases of blood clots associated with thrombocytopenia, i.e., low levels of blood platelets with or without bleeding, including rare cases of cerebral venous thrombosis (CVT). We report the case of a 54-year-old woman who developed disseminated intravascular coagulation (DIC) with multi-district thrombosis 12 days after the AstraZeneca COVID-19 vaccine administration. A brain computed tomography (CT) scan showed multiple subacute intra-axial hemorrhages in atypical locations, including the right frontal and the temporal lobes. A plain old balloon angioplasty (POBA) of the right coronary artery was performed, without stent implantation, with restoration of distal flow, but with persistence of extensive thrombosis of the vessel. A successive thorax angio-CT added the findings of multiple contrast filling defects with multi-vessel involvement: at the level of the left upper lobe segmental branches, of left interlobar artery, of the right middle lobe segmental branches and of the right interlobar artery. A brain magnetic resonance imaging (MRI) in the same day showed the presence of an acute basilar thrombosis associated with the superior sagittal sinus thrombosis. An abdomen angio-CT showed filling defects at the level of left portal branch and at the level of right suprahepatic vein. Bilaterally, it was adrenal hemorrhage and blood in the pelvis. An evaluation of coagulation factors did not show genetic alterations so as the nasopharyngeal swab ruled out a COVID-19 infection. The patient died after 5 days of hospitalization in intensive care.

## 1. Introduction

In December 2019, health Chinese authorities recognized a cluster of acute respiratory disease of unknown etiology [1]. Afterwards, a new viral agent, SARS-CoV-2, was isolated as responsible for the heart of an epidemic located in Hubei. On 30 January 2020, the World Health Organization (WHO) defined the COVID-19 epidemic as a public health emergency and on 11 March 2020 as a pandemic in the world [1,2]. Currently, a valuable therapy has not yet been improved so that mechanical respiratory support is the only treatment in critically ill patients [3,4,5]. In this scenario, it was essential to develop a vaccine as soon as possible, to prevent SARS-CoV-2 and to protect persons who are at high risk for complications. At the time of writing three vaccines have been approved in Italy: the mRNA-1273 vaccine Moderna [6], the mRNA BNT162b2 Pfizer drug [7], and the ChAdOx1 nCoV-19 vaccine (AZD1222), that consists of a replication-deficient chimpanzee adenoviral vector ChAdOx1, containing the SARS-CoV-2 structural surface glycoprotein antigen (spike protein; nCoV-19) gene [8]. Globally, at the time of writing (20 March 2021), 121.759.109 confirmed COVID-19 cases have been reported to WHO, including 2.690.731 deaths. Globally, on 18 March 2021, a total of 364.184.603 vaccine doses have been administered. In Italy, 3.306.711 confirmed COVID-19 cases with 103.855 deaths have been reported to WHO. In Italy, on 9 March 2021, a total of 6.634.450 vaccine doses have been administered [2].

The Italian authorities have used the following vaccination strategy, reserving the mRNA vaccines for health personnel and the population over 80 years old, and the administration of the COVID-19 Vaccine AstraZeneca for law enforcement personnel, school personnel and the population between 70 and 80 years old [9]. A strategy for monitoring adverse events of vaccines is based on the collaboration of local and national health structures, assisted by Italian Medicines Agency (AIFA) [10,11]. On 15 March 2021, AIFA has decided to suspend temporarily the use of the AstraZeneca Covid19 vaccine throughout the country as a precaution, pending the rulings of the European Medicines Agency (EMA). This decision was taken in line with similar measures adopted by other European countries due to the death of some vaccinated people [10]. On 18 March 2021, EMA’s safety committee concluded its preliminary review about thromboembolic events in people vaccinated with COVID-19 Vaccine AstraZeneca at its extraordinary meeting [12]. The Committee confirmed that:

- the benefits of the vaccine in combating the still widespread threat of COVID-19 (which itself results in clotting problems and may be fatal) continue to outweigh the risk of side effects;
- the vaccine is not associated with an increase in the overall risk of blood clots (thromboembolic events) in those who receive it;
- there is no evidence of a problem related to specific batches of the vaccine or to particular manufacturing sites;
- however, the vaccine may be associated with very rare cases of blood clots associated with thrombocytopenia, i.e., low levels of blood platelets with or without bleeding, including rare cases of cerebral venous thrombosis (CVT).

Rare cases—around 20 million people in the UK and European Economic Area (EEA) received the vaccine until 16 March and the EMA reviewed only 7 cases of blood clots in multiple blood vessels (disseminated intravascular coagulation (DIC)) and 18 cases of CVT. A causal link with the vaccine is not proven but is possible and it deserves further analysis [12].

We report the case of a woman who developed DIC with multi-district thrombosis 12 days after the AstraZeneca COVID-19 vaccine administration.

## 2. Case

The study was conducted according to the guidelines of the Declaration of Helsinki. Approval by the Institutional Review Board was not needed considering the nature of the study: the findings description of a single case report. Informed consent was obtained by the patient. On 13 March 2021, a 54-year-old woman patient arrived at the emergency room at 10.00 a.m. because of an acute cerebrovascular accident. In anamnesis a Meniere’s disease and recent administration of the AstraZeneca vaccine (12 days ago) was reported, without any kind of drug therapy. At clinical evaluation left side signs were found, with a Glasgow Coma Score (GCS) 13; the lower limbs were normothermic and normoconformed, with the preserved femoral, popliteal, and posterior tibial wrist bilaterally. Laboratory tests showed elevated cardiac enzymes, PT 51%, PTT 41 sec, elevated D dimers and normal fibrinogen with blood count, normocytic anemia (HB 8.7 g/dL) and thrombocytopenia, signs of DIC. ECG showed signs of myocardial infarction. Ecocolordoppler examination excluded deep vein thrombosis (DVT) in the explored vessels, with patency of the distal popliteal femoral arterial axis with normal flows.

Clinical and laboratory tests excluded sepsis, various infections, malignancy, vascular diseases, toxic and immunological reactions.

Evaluation of coagulation factors did not show genetic alterations so as the nasopharyngeal swab ruled out a COVID-19 infection.

No severe trauma was reported.

**Figure 1:**
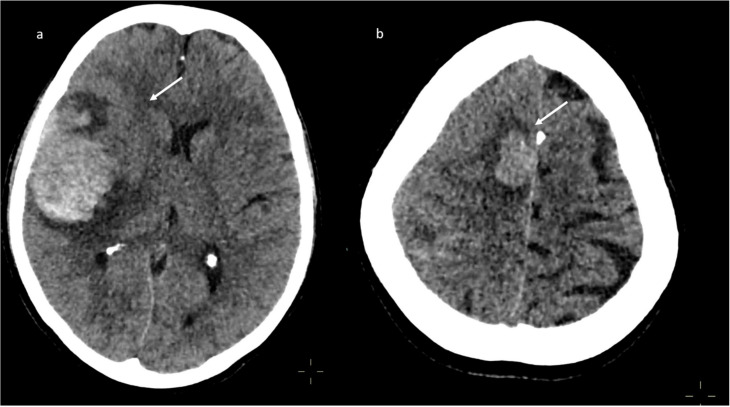
Brain computed tomography (CT) scan: presence of multiple subacute intra-axial hemorrhages in atypical locations (**a**,**b**).

**Figure 2:**
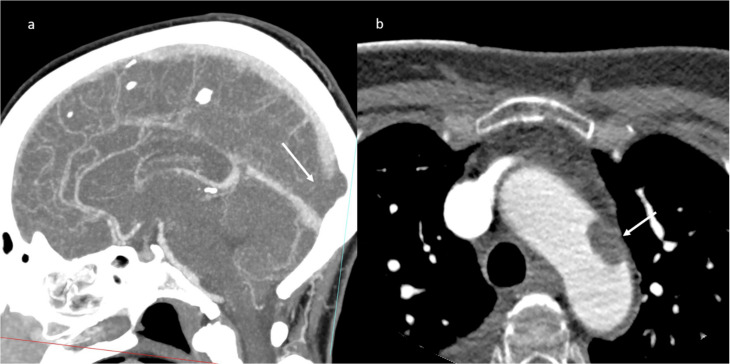
CT-angiography: partial thrombosis of the vein of Galen (**a**); presence of a floating thrombus within the aortic arch (**b**).

A brain computed tomography (CT) scan showed multiple subacute intra-axial hemorrhages in atypical locations, including the right frontal and the temporal lobes (Figure 1), with ipsilateral hemorrhagic subarachnoid suffusion, raising the suspicion of Labbè/superior longitudinal sinus thrombosis, even if brain angio-CT demonstrated only a non-occlusive thrombosis of the vein of Galen (Figure 2a), but also a floating thrombus within the aortic arch (Figure 2b).

**Figure 3:**
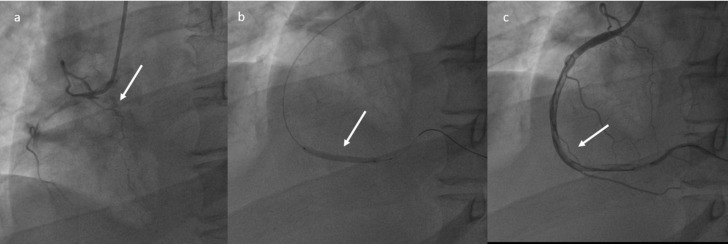
Plain Old Balloon Angioplasty (POBA) of the right coronary artery (**a**–**c**): restoration of the distal flow, with persistence of extensive thrombosis.

The patient was transferred to the Hemodynamics room at 1.00 p.m. a plain old balloon angioplasty (POBA) of the right coronary artery was performed, without stent implantation, with restoration of distal flow, but with persistence of extensive thrombosis of the vessel (Figure 3) with a theoretic indication for administration of intracoronary antiplatelet agents (Aggrastat). However, given the hematological and neurological status, such therapy was not performed. A progressive worsening of the neurological state was observed until a comatose status (GCS 6) started during the procedure. The patient was transferred to the Intensive Care Unit.

**Figure 4:**
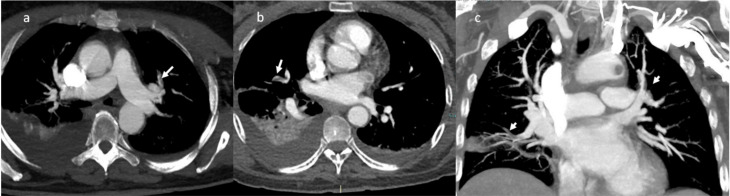
CT-angiography: presence of multiple contrast filling defects involving the left upper lobe segmental branches (**a**), the right segmental artery (**b**); MPR coronal plane in (**c**).

A successive thorax angio-CT added the findings of multiple contrast filling defect with multi-vessel involvement: at the level of the left upper lobe segmental branches, of left interlobar artery, of the right middle lobe segmental branches and of the right interlobar artery (Figure 4).

**Figure 5:**
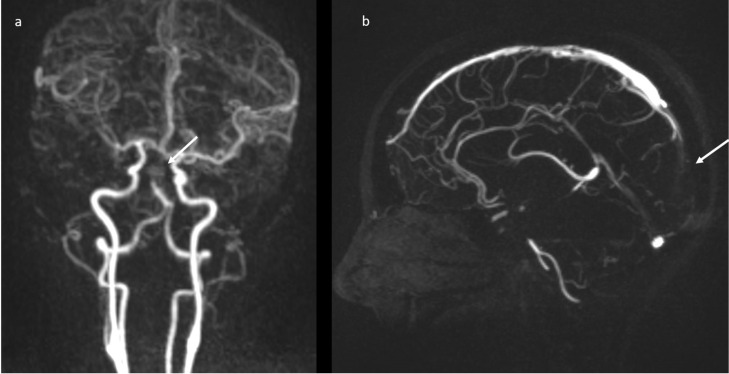
MR-angiography: acute basilar thrombosis associated with superior coronal (**a**) and sagittal (**b**) sinus thrombosis.

**Figure 6:**
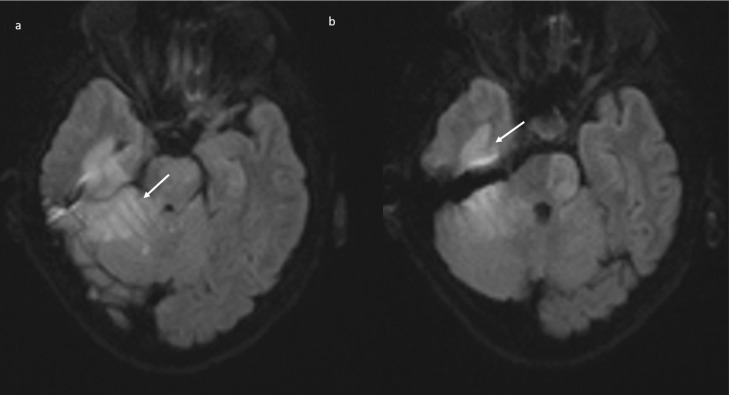
Brain MRI (DWI): acute ischemic lesion with restricted diffusion involving the pons, mesencephalon, the right superior cerebellar hemisphere with the vermis (**a**), and the right posterior temporal lobe (**b**).

Brain magnetic resonance imaging (MRI) in the same day showed the presence of an acute basilar thrombosis (Figure 5a) associated with the superior sagittal sinus thrombosis (Figure 5b) with the delineation of hyperacute ischemic lesions in the vascular territory of the right posterior cerebral artery and of the perforating pontine branches (Figure 6).

**Figure 7:**
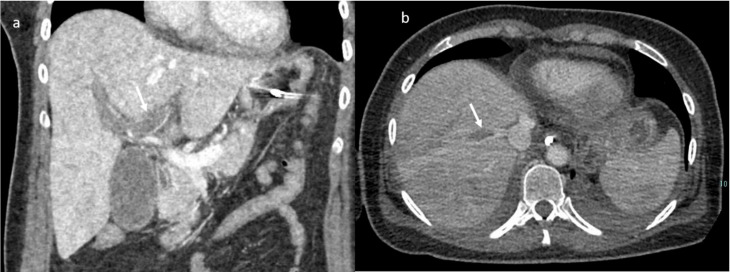
CT scan; in (**a**) MPR (arrow shows thrombosis of the left portal branch) and in (**b**) axial plain during portal phase arrow shows thrombosis if the right (Figuer suprahepatic vein).

**Figure 8:**
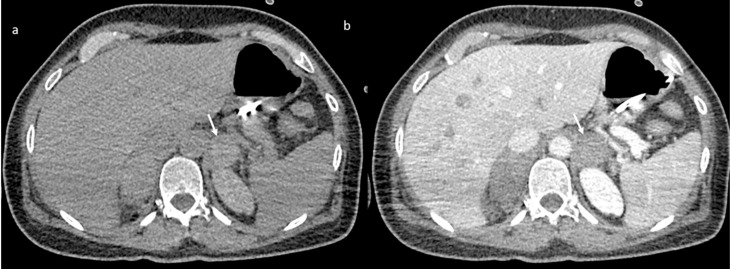
CT scan without (**a**) and with contrast (**b**) shows adrenal hemorrhage (arrow).

An abdomen angio-CT showed filling defects at the level of left portal branch (Figure 7) and at the level of right suprahepatic vein (7). Bilaterally, it was adrenal hemorrhage (Figure 8) and blood in the pelvis.

**Figure 9:**
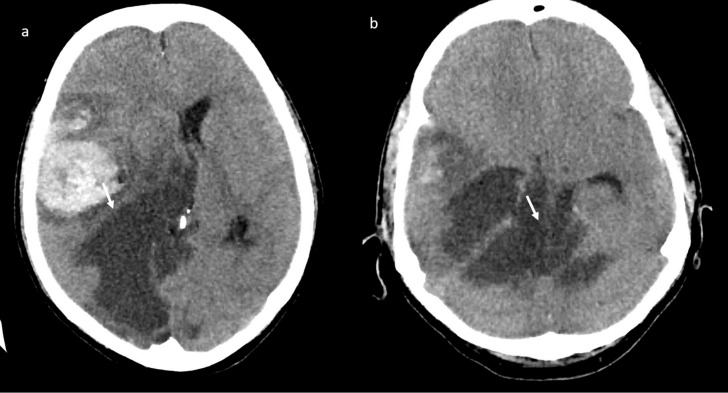
Brain CT scan: diffuse ischemic hypodensity involving the right occipito-temporal (**a**) and the superior cerebellar regions, the right thalamic and internal capsula regions, pons and mesencephalon (**b**).

A brain CT performed one day later showed a diffuse ischemic hypodensity involving the right occipito-temporal and the superior cerebellar regions, the right thalamic and internal capsula regions, pons, and mesencephalon (Figure 9), conditioning edema-based mass effect and contralateral shift of the midline structures.

The patient died after 5 days of hospitalization in intensive care.

## 3. Discussion

The efficacy and safety of the ChAdOx1 nCoV-19 vaccine includes data from four ongoing blinded, randomized, controlled trials done across three countries: COV001 (phase 1/2; UK), COV002 (phase 2/3; UK), COV003 (phase 3; Brazil), and COV005 (phase 1/2; South Africa). The interim efficacy is assessed by a prespecified global pooled analysis combining data from COV002 and COV003. The safety of the vaccine is assessed using data from all four studies [8]. Three of the studies are single blind and one is double blind (COV005). Primary efficacy was assessed in participants who received two doses of the vaccine. All four studies included participants who received two doses, with a booster dose incorporated into the three trials, that were initially designed to assess a single-dose of ChAdOx1 nCoV-19 compared with control (COV001, COV002, and COV003) after review of the antibody response data from COV001 [8]. All four studies stated that the vaccine has a good safety profile with serious adverse events and adverse events of special interest balanced across the study arms. Serious adverse events occurred in 168 participants, 79 of whom received ChAdOx1 nCoV-19 and 89 of whom received MenACWY or saline control [8]. A total of 175 events were reported (84 in the ChAdOx1 nCoV-19 group and 91 in the control group), three of which were considered possibly related to either the experimental or a control vaccine. A case of transverse myelitis was reported 14 days after ChAdOx1 nCoV-19 booster vaccination possibly related to vaccination. A potentially vaccine-related serious adverse event was reported 2 days after vaccination in South Africa in an individual who recorded fever higher than 40 °C, but who recovered rapidly without an alternative diagnosis and was not admitted to the hospital. Four non-COVID-19 deaths were reported from the studies (three in the control arm and one in the ChAdOx1 nCoV-19 arm), all considered unrelated to the vaccine [8]. Nevertheless, due to the occurrence of some episodes of DIC and CVT, on 15 March 2021, European countries decided to suspend temporarily the use of the AstraZeneca Covid19 vaccine throughout the country as a precaution, pending the rulings of the EMA. According to the EMA data, around 20 million people in the UK and EEA had received the ChAdOx1 nCoV-19 vaccine as of March 16 and EMA had reviewed only 7 cases of blood clots in multiple blood vessels (disseminated intravascular coagulation, DIC) and 18 cases of CVST. A causal link with the vaccine is not proven but is possible and deserves further analysis [12]. The EMA Pharmacovigilance Risk Assessment Committee (PRAC) involved experts in blood disorders in its review and worked closely with other health authorities including the UK’s MHRA which has experience with administration of this vaccine to around 11 million people. Overall, the number of thromboembolic events reported after vaccination, both in studies before licensing and in reports after rollout of vaccination campaigns (469 reports, 191 of them from the EEA), was lower than that expected in the general population [8]. This allows the PRAC to confirm that there is no increase in overall risk of blood clots. However, in younger patients there remain some concerns, related in particular to these rare cases. The Committee’s experts looked in extreme detail at records of DIC and CVST reported from Member States, 9 of which resulted in death. Most of these occurred in people under 55 and the majority were women [12]. Because these events are rare, and COVID-19 itself often causes blood clotting disorders in patients, it is difficult to estimate a background rate for these events in people who have not had the vaccine. In fact, besides the respiratory manifestations with severe disabling complications, another major concern is represented by evidence of consistent hemostatic changes in patients with severe or critical COVID-19, likely related to a pro-thrombotic switch [13,14,15,16,17,18,19,20,21,22,23,24,25,26,27,28,29,30,31,32]. Among COVID-19 patients, it is reasonable to assume that those with a very severe disease could exhibit high risk of venous thromboembolism (VTE), including deep vein thrombosis (DVT) and/or pulmonary embolism (PE) [31]. In recently published studies, the incidence of PE in patients with coronavirus disease 2019 (COVID-19) who underwent pulmonary CT angiography was reported to be between 23% and 30% [14,15] and the severity of COVID-19 infection should be an important feature in the onset of PE in critically ill patients [14,15]. However, based on pre-COVID-19 figures, it was calculated that less than 1 reported case of DIC might have been expected by 16 March among people under 50 within 14 days of receiving the vaccine, whereas 5 cases had been reported. Similarly, on average 1.35 cases of CVST might have been expected among this age group whereas by the same cut-off date there had been [12]. A similar imbalance was not visible in the older population given the vaccine [12].

We reported the case of a woman who developed a multidistrict thrombotic condition 12 days after AstraZeneca COVID-19 vaccine administration. Although it is not possible to establish the causal link with the vaccine, it should nevertheless be emphasized that the patient had no concomitant cause of DIC.

DIC is defined as a condition characterized by systemic intravascular activation of coagulation, leading to the widespread deposition of fibrin, with formation of widespread microvascular thrombosis. During the coagulation process, consumption of coagulation factors and aggregation of platelets occur resulting in reduced levels of both procoagulant and anticoagulant clotting proteins. Therefore, thromboembolic events and hemorrhage may coexist [32,33]. The uniqueness of the case herein reported relies on the widespread arterial and venous large vessel involvement, with polidistrict organ dysfunction related to the predominance of thrombus formation. To the best of our knowledge this is the first case described in the literature in which a temporal relationship with the administration of a vaccine is found. DIC is not a disease by itself, but always secondary to an existing disease. It may occur especially after sepsis, various infections, malignancy, obstetric and vascular diseases, severe trauma, toxic and immunological reactions [34,35,36]. Severe anaphylaxis and hemolytic transfusion reaction are associated to DIC [34]. A variety of relevant mechanisms contributing to the derangement of coagulation in DIC have been elucidated. Initiation and propagation of coagulation with concurrent impairment of physiological anticoagulant pathways and a deficit of endogenous fibrinolysis, all as a result of systemic inflammatory activation, are resulting in platelet activation and fibrin deposition. Important inflammatory mediators that govern these processes include tumor necrosis factor (TNF)-α and interleukin (IL)-1 and IL-6. In addition, recent work indicates that intravascular webs (“neutrophil extracellular traps”) composed of denatured DNA from destructed cells and entangling neutrophils, platelets, fibrin, and cationic proteins, such as histones, may play a crucial role in the development of thrombus deposition [34].

In this reported case there is no evidence of sepsis, as well as no genetic alterations of coagulation or recent trauma or malignancy. The only factor showing a temporal association was vaccine administration.

## 4. Conclusions

According to the EMA Committee, the vaccine’s proven efficacy in preventing hospitalization and death from COVID-19 outweighs the extremely small likelihood of developing thromboembolic events. However, we should be aware of the remote possibility of such events. In case of suggestive symptoms immediate medical attention is needed, informing healthcare professionals of the recent vaccination. Close safety monitoring of reports of blood clotting disorders will continue, and further studies are being instituted to provide more laboratory data as well as real-world evidence.

## Data Availability

All data are presented in the manuscript.
